# Clinical and molecular characteristics of *Streptococcus agalactiae* bacteremia in nonpregnant adults: a single-center analysis over 16 years in Hiroshima, Japan

**DOI:** 10.1007/s10096-026-05520-6

**Published:** 2026-04-30

**Authors:** Hiroki Kitagawa, Satoshi Nakano, Kayoko Tadera, Yuta Kuhara, Keitaro Omori, Norifumi Shigemoto, Shota Koide, Shogo Otake, Kasumi Ishida-Kuroki, Yo Sugawara, Motoyuki Sugai, Hiroki Ohge

**Affiliations:** 1 Department of Infectious Diseases, Hiroshima University Hospital, Hiroshima, Japan; 2 Department of Surgery, Graduate School of Biomedical and Health Sciences, Hiroshima University, Hiroshima, Japan; 3 Antimicrobial Resistance Research Center, National Institute of Infectious Diseases, Japan Institute for Health Security, Tokyo, Japan; 4 Section of Clinical Laboratory, Division of Clinical Support, Hiroshima University Hospital, Hiroshima, Japan; 5 Division of Laboratory Medicine, Hiroshima University Hospital, Hiroshima, Japan; 6 Translational Research Center, Hiroshima University, Hiroshima, Japan

**Keywords:** Group B *Streptococcus*, Bacteremia, Streptococcal toxic shock syndrome, Whole genome sequencing, Septic shock

## Abstract

**Purpose:**

Molecular epidemiological data on *Streptococcus agalactiae* (Group B *Streptococcus* [GBS]) causing bacteremia in non-pregnant adults in Japan remain limited. This study elucidated the clinical characteristics and molecular epidemiology of GBS strains causing bacteremia in this population.

**Methods:**

Data on non-pregnant adults with GBS treated at Hiroshima University Hospital between 2008 and 2023 were retrospectively reviewed. Clinical data were collected, and strains were characterized for antimicrobial susceptibility by susceptibility testing and whole-genome sequencing.

**Results:**

A total of 73 adult patients with 74 episodes of GBS bacteremia were identified. The most common infection sites were skin and soft tissue (25.7%) and bacteremia with unknown focus (20.3%). Streptococcal toxic shock syndrome (STSS) was observed in 17 patients (23.0%), and the 30-day mortality rate was 25.7%. The predominant serotype/clonal complex (CC) lineages were Ib/CC12 (*n* = 14, 18.9%), V/CC1 (*n* = 12, 16.2%), and Ia/CC23 (*n* = 7, 9.5%). Notably, all seven Ia/CC23 strains and 10 (83.3%) V/CC1 strains were recovered after 2016. Single-nucleotide polymorphism-based analysis revealed the presence of strains within the ST17 and ST464 lineages circulating between pediatric and adult populations.

**Conclusions:**

This study demonstrates the emergence of the Ia/CC23 and V/CC1 lineages among invasive GBS strains causing bacteremia in non-pregnant adults in Japan over the past 10 years. Identifying the transmission pathways linking different age groups may help reduce the burden of invasive GBS infection in both pediatric and adult populations and develop effective public health and preventive strategies.

**Supplementary Information:**

The online version contains supplementary material available at 10.1007/s10096-026-05520-6.

## Introduction

*Streptococcus agalactiae* (Group B *Streptococcus* [GBS]) is a major cause of invasive disease in neonates and pregnant women [1, 2]. Although historically considered a perinatal pathogen, GBS has increasingly been recognized as an important cause of invasive infections among non-pregnant adults worldwide, particularly among the elderly and individuals with underlying comorbidities [3]. Population-based surveillance conducted by the Active Bacterial Core surveillance network identified 21,250 non-pregnant adults who developed invasive GBS infection (iGBS) in the United States between 2008 and 2016 [4]. During the study period, the incidence of iGBS significantly increased from 8.1 to 10.9 cases per 100,000 individuals. The incidence progressively increased with advancing age, peaking among individuals aged ≥ 80 years, who accounted for 17.7% of all patients. The overall case fatality rate was 6.5%, declining from 7.5% in 2008 to 5.6% in 2016. Additionally, the proportion of patients with iGBS presenting at least one underlying condition increased from 90.7% in 2008 to 94.6% in 2016 (*p* = 0.005 for trend).

Since its initial description by Stevens [5], streptococcal toxic shock syndrome (STSS) has been widely recognized as a severe clinical manifestation of *Streptococcus pyogenes* (group A *Streptococcus*) infection. Against this conceptual background, severe infections caused by other β-hemolytic *Streptococcus* species (BHS) are sometimes classified as “invasive/fulminant streptococcal infections” and are often considered STSS-like, even in the absence of clinical evidence implicating superantigen-mediated mechanisms. In Japan, in accordance with the diagnostic criteria for STSS established by the United States Centers for Disease Control and Prevention in 2010 [6], both severe GAS infections and infections caused by other *Streptococcus* species, including groups B, C, and G, are designated as notifiable diseases. Physicians diagnosing these conditions are legally required to report such cases to local public health authorities.

Consequently, although epidemiological data are available in Japan regarding the involvement of iGBS in symptom-based STS-like syndromes, such as its relative frequency compared with other Lancefield group streptococci, the overall mortality rates among patients with iGBS remain limited. Currently, maternal vaccines targeting GBS are under active development [7, 8]. Similar to the pneumococcal vaccines, these vaccines may eventually be extended to adults. In this context, robust epidemiological data on the incidence and mortality of iGBS in adults are essential to accurately estimate the cost-effectiveness of such vaccination strategies.

A recent systematic review demonstrated substantial molecular diversity among GBS strains from non-pregnant adults, with pronounced geographic variation in dominant clonal complex (CC) and antimicrobial resistance profiles [3]. In Japan, several regional and nationwide investigations have characterized the molecular features of iGBS strains; however, most studies have focused on infants or mixed-age cohorts, and comprehensive molecular epidemiological data specific to non-pregnant adults remain limited [9–11]. Furthermore, Kasai et al.. recently demonstrated that in pediatric iGBS, closely related strains were detected across broad temporal and spatial ranges, suggesting the circulation of pediatric GBS strains within the environment [12]. However, to date, no study has examined the relationship between strains derived from pediatric iGBS and those detected in non-pregnant adults.

In this study, clinical data and whole-genome sequencing information of strains from adult patients with iGBS who received treatment over approximately 15 years at a single teaching hospital were analyzed to elucidate the association between the clinical characteristics of adult iGBS in Japan and the bacterial traits of the causative strains. Furthermore, by comparing the genomic data of the strains collected in this study with those of previously reported pediatric iGBS strains in Japan, we explored possible epidemiological links between adult and pediatric populations.

## Materials and methods

### Study design and participants

This retrospective cohort study included all adult patients with iGBS who were admitted to Hiroshima University Hospital between January 1, 2008, and December 31, 2023. All the data on the GBS strains isolated from blood cultures during the study period were retrieved from the hospital’s laboratory information system. Only patients aged ≥ 18 years were enrolled in the study. Women who were pregnant or delivered within 30 days before bacterial isolation were excluded. Patients who experienced recurrent episodes of bacteremia during the same hospitalization were also excluded. Episodes that met the above inclusion criteria were treated as independent events, even when they occurred in the same patient. This study was approved by the Ethics Committee for Epidemiology of Hiroshima University (E2020-2133-01) and the National Institute of Infectious Diseases (submission ID: 1512). The requirement for informed consent was waived because of the non-invasive nature of the study.

### Collection of clinical data

Clinical data, including patient demographics, comorbidities, Charlson Comorbidity Index [13], source of bacteremia, presence of septic shock, occurrence of STSS at the time of blood culture collection, blood culture results, and 28-d mortality rate, were collected from the hospital’s electronic medical system. Septic shock was defined as previously described [14]. STSS was diagnosed based on the following three criteria [6]: (1) an infection caused by the previously mentioned BHS; (2) hypotension, defined as a systolic blood pressure of ≤ 90 mm Hg; and (3) multiorgan involvement, defined as the presence of two or more of the following: renal impairment (creatinine ≥ 2 mg/dL or a > 2-fold increase over the baseline level in patients with preexisting renal disease), coagulopathy (platelet count ≤ 100,000 cells/µL or evidence of disseminated intravascular coagulation), hepatic involvement (alanine aminotransferase, aspartate aminotransferase, or total bilirubin levels ≥ 2 times the upper limit of normal for the patient’s age, or a 2-fold increase over the baseline level in patients with preexisting liver disease), acute respiratory distress syndrome, or a generalized erythematous macular rash or soft tissue necrosis. The diagnoses of septic shock and STSS were subsequently confirmed through a retrospective review of medical records by two infectious disease physicians.

### Microbiological procedure

Blood culture was performed using the BacT/ALERT 3D system (bioMérieux, Marcy l’Étoile, France) from January 1, 2008, to April 17, 2022, and using the BacT/ALERT Virtuo system (bioMérieux) from April 18, 2022, to December 31, 2023. Bact/ALERT FA and FN Plus bottles (bioMérieux) were used during both periods. Bacterial species were identified using the Vitek 2 compact system with a Vitek 2 GP ID card (bioMérieux) from January 1, 2016, to March 31, 2021, and matrix-assisted laser desorption/ionization time-of-flight mass spectrometry with a MALDI Biotyper Sirius system (Bruker Daltonik GmbH, Bremen, Germany) from April 1, 2021, to December 31, 2023. GBS strains were stored in Microbank™ (Pro Lab Diagnostics Inc., Richmond Hill, Canada) at − 80 °C until further use.

### Antimicrobial susceptibility testing and serotyping

Antimicrobial susceptibility testing was performed using the broth microdilution method with IA40 MIC-i Dry Plates (Eiken Chemical Co., Ltd., Tokyo, Japan) following the Clinical and Laboratory Standards Institute (CLSI) reference standards [15]. The minimum inhibitory concentrations of penicillin (PEN), cefotaxime (CTX), erythromycin (ERY), clindamycin (CLI), and levofloxacin (LVX) were determined. In addition, we assessed inducible CLI resistance using wells containing a combination of 0.5 µg/mL CLI and 1 µg/mL ERY [15]. Susceptibility categories—susceptible (S), intermediate (I), and resistant (R)—were assigned according to the CLSI 2024 guidelines. For antimicrobials lacking CLSI-defined I or R categories, strains not classified as “S” were considered “R” for consistency.

Serotyping was performed using the GBS latex agglutination test kit, ImmuLex Strep-B Kit (SSI Diagnostica, Hillerød, Denmark), according to the manufacturer’s instructions.

### Whole-genome sequencing

Genomic DNA was extracted using the QIAamp^®^ DNA Mini Kit (QIAGEN, Hilden, Germany) according to the manufacturer’s instructions. Multiplexed samples were sequenced on an Illumina NovaSeq X Plus platform for 300 cycles, generating 150 bp paired-end reads. After trimming and quality control, the data were analyzed using an in-house pipeline [16]. Briefly, in silico multilocus sequence typing [17] was performed using mlst (https://github.com/tseemann/mlst), and resistance genes were identified using ABRicate version 1.0.1 (https://github.com/tseemann/abricate) with the National Center for Biotechnology Information (NCBI) [18] and the ResFinder database [19]. Surface proteins, including hypervirulent GBS adhesin (HvgA), Srr proteins (Srr1 and Srr2), alpha protein family members (BCA, Rib, Alp2/3, and Alp1), and pilus islands (PI-1, PI-2 A, and PI-2B) were detected as previously described [20]. A k-mer-based maximum likelihood phylogenetic tree was constructed for all tested strains using kSNP4 v4.1 [21]. To identify potential endemic GBS subclusters, single-nucleotide polymorphism (SNP) analysis using the CFSAN SNP Pipeline version 2.2.1 [22] was performed on strains within each sequence type and compared with those from invasive pediatric iGBS in Japan between 2004 and 2023 [12]. In this comparison, we defined strains with ≤ 15 pairwise SNPs as having genetic linkage, based on the total number of SNPs (14 SNPs per year) reported to accumulate over one year in the whole-genome analysis of the GBS-ST283 outbreak strain that occurred in Singapore in 2015 [23]. In addition, strains differing by 16–20 pairwise SNPs were arbitrarily classified as showing potential genetic linkages.

### Statistical analysis

Categorical variables were expressed as frequencies and percentages (%), whereas continuous variables were expressed as medians with interquartile ranges. Categorical variables were compared using Fisher’s exact test. Statistical significance was set at *p* < 0.05. All analyses were conducted using the JMP Pro software (version 18.0; SAS Institute Inc., Cary, NC, USA).

## Results

### Patient characteristics and clinical manifestations

During the study period, 73 adult patients experienced 74 episodes of iGBS. The patient demographics and clinical characteristics are summarized in Table 1. The median age was 69 years (interquartile range [IQR]: 58–77 years), and 47 (63.5%) patients were aged ≥ 65 years. The study cohort included 48 male patients (64.8%). The most common comorbidity was solid organ malignancy (37.8%), followed by diabetes mellitus (36.5%) and liver disease (24.3%). The primary sources of iGBS were skin and soft tissue infections (25.7%), bacteremia without an identifiable focus (20.3%), and catheter-related bloodstream infections (9.5%). Thirteen patients developed polymicrobial bacteremia, with *Staphylococcus aureus* being the most frequently co-isolated species (53.8%). STSS was observed in 23% of patients, and the 30-day mortality rate was 25.7%.

**Table 1:** Demographics and clinical characteristics of non-pregnant adult patients with GBS bacteremia

Variable	Total, *n* = 74
Age, median (IQR), years	69.5 (58.0-77.3)
Age > 65 years, n (%)	47 (63.5)
Male, n (%)	48 (64.9)
Comorbidities [n (%)]	
Solid-organ malignancy	28 (37.8)
Diabetes mellitus	27 (36.5)
Liver diseases	18 (24.3)
Cardiovascular failure	16 (21.6)
Kidney diseases	11 (14.9)
Hematologic malignancy	6 (8.1)
Chronic pulmonary diseases	6 (8.1)
Charlson Comorbidity Index, median (IQR)	2.5 (1–5)
Charlson Comorbidity Index > 2	37 (50)
Source of bacteremia, n (%)	
Skin and soft tissue infections	19 (25.7)
Bacteremia without primary focus	15 (20.3)
Catheter-related bloodstream infections	7 (9.5)
Urinary tract infections	6 (8.1)
Intra-abdominal infections	5 (6.8)
Bone and joint infections	5 (6.8)
Respiratory tract infections	5 (6.8)
Infective endocarditis	5 (6.8)
Genital tract infections	2 (2.7)
Central nervous system infections	1 (1.4)
Others	4 (5.4)
Polymicrobial bacteremia	13 (17.6)
Bacteria species identified with *S. agalactiae* in blood cultures	
* Staphylococcus aureus*	7 (9.5)
* Escherichia coli*	4 (5.4)
* Enterococcus faecium*	1 (1.4)
* Aeromonas sobria*	1 (1.4)
* Serratia marcescens*	1 (1.4)
* Corynebactetrium* sp.	1 (1.4)
* Proteus mirabilis*	1 (1.4)
Others	2 (2.7)
Septic shock, n (%)	27 (36.5)
Streptococcal toxic shock syndrome, n (%)	17 (23.0)
30 days mortality, n (%)	19 (25.7)

### Serotype and multi-locus sequence typing

**Fig. 1:**
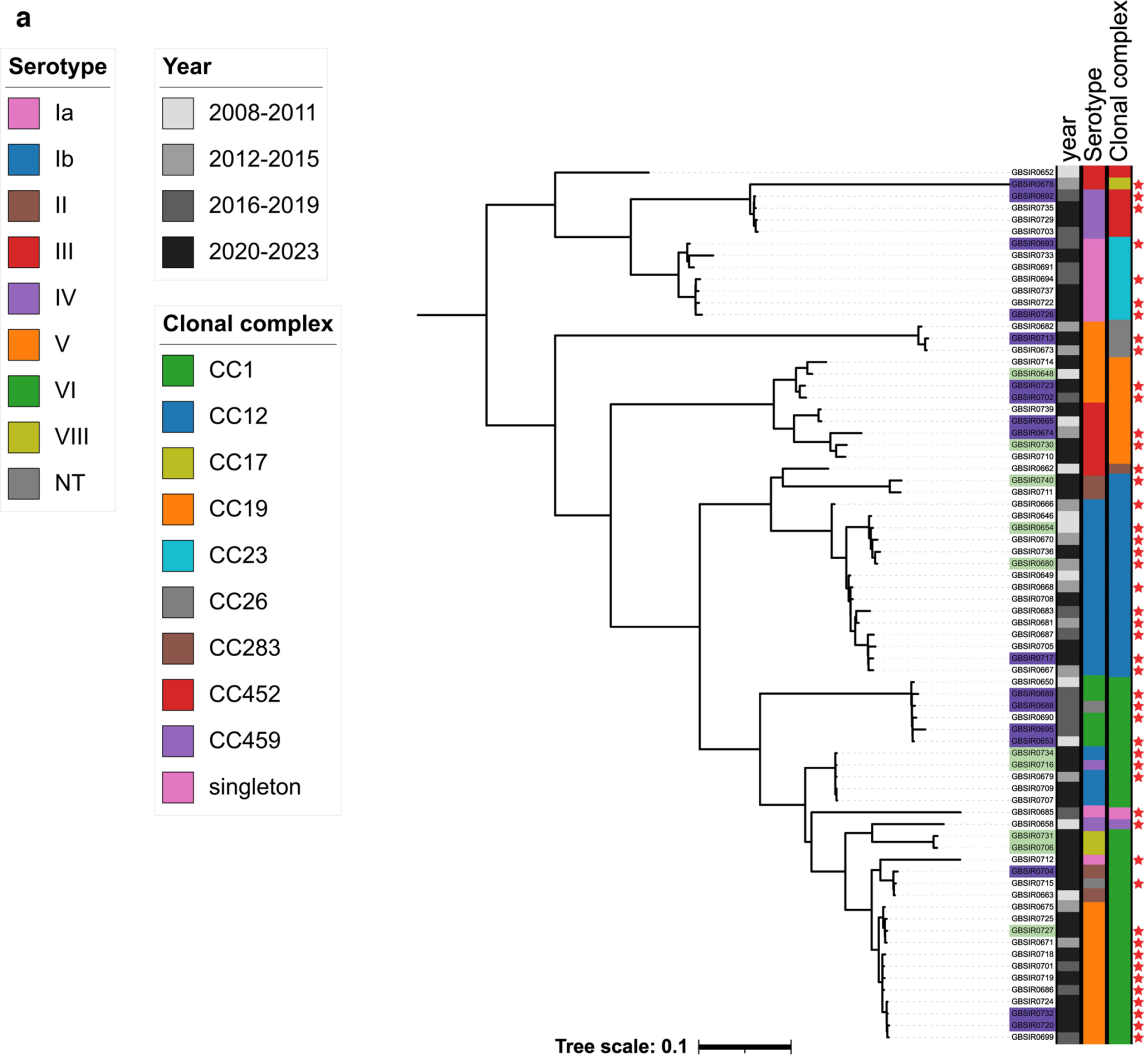

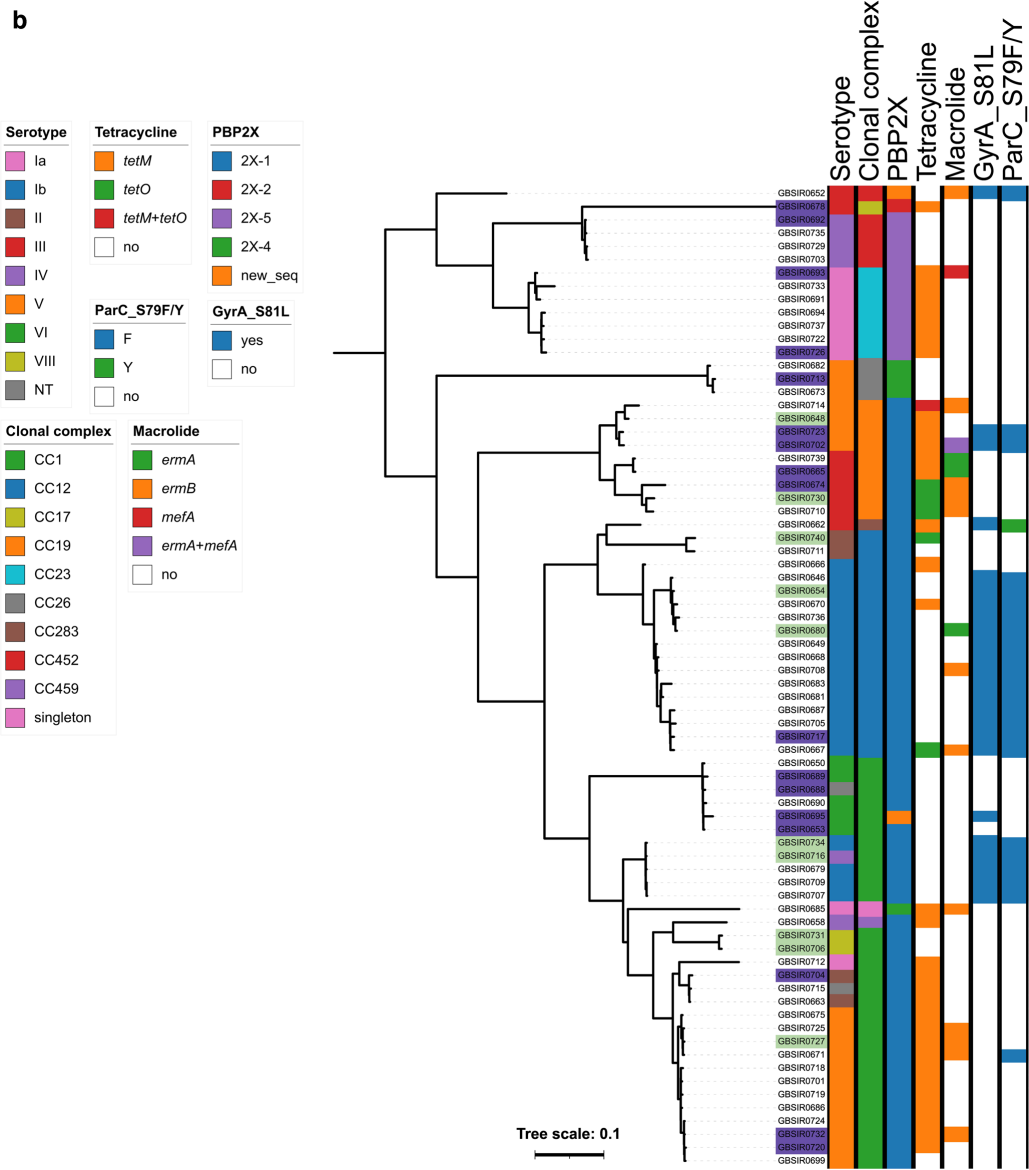

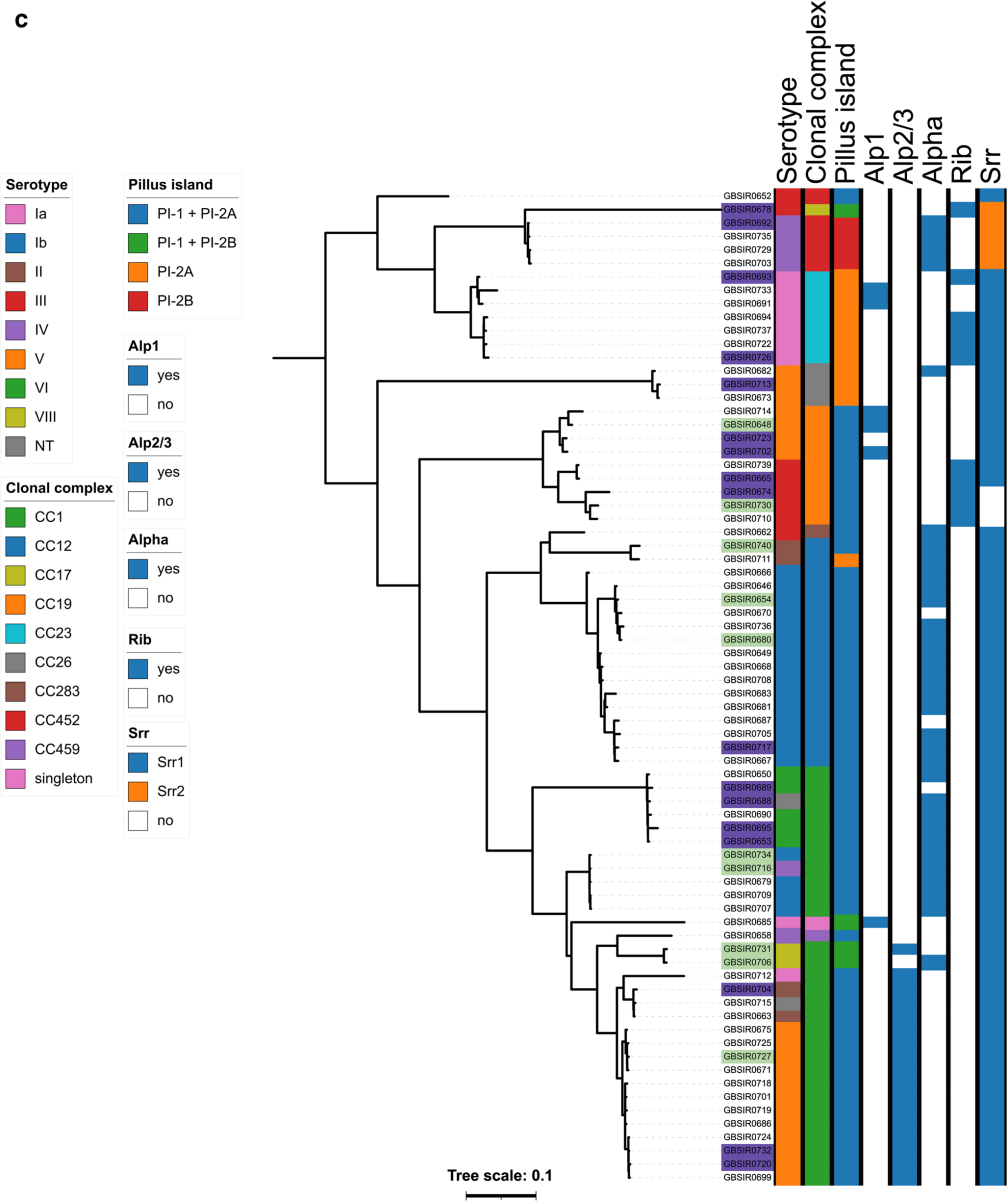
Maximum likelihood phylogenetic tree of the 74 *Streptococcus agalactiae* strains analyzed in this study. (**A**) Phylogenetic tree annotated with metadata on serotype, genotype (clonal complex), and year of isolation. Clinical syndrome is indicated, with light green sample names representing septic shock and purple indicating STSS. Red stars (★) denote patients aged ≥65 years. (**B**) Phylogenetic tree annotated with metadata on serotype, genotype (clonal complex), and resistance genes. (**C**) Phylogenetic tree annotated with metadata on serotype, genotype (clonal complex), and major candidate GBS vaccine targets.

**Fig. 2:**
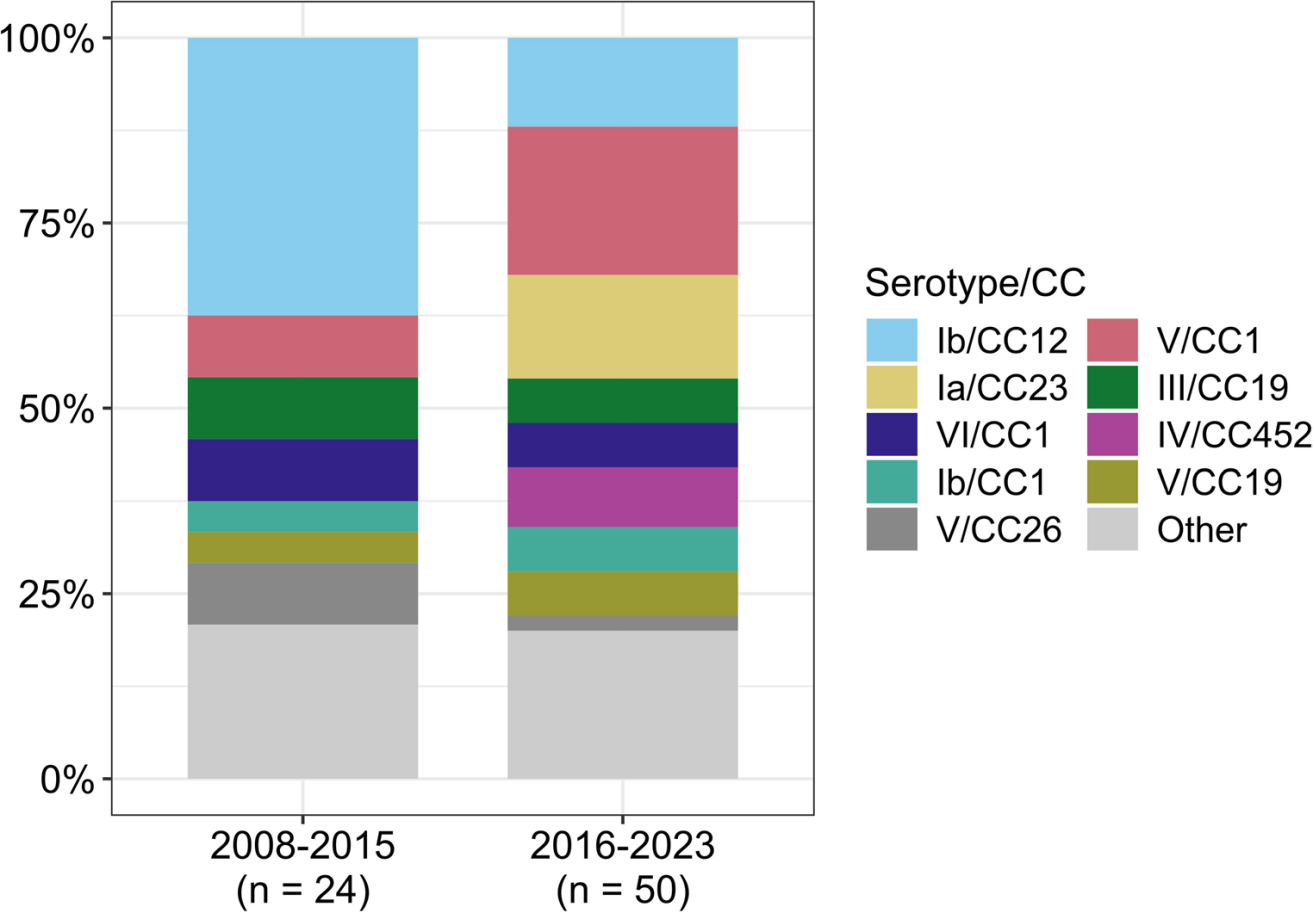
Bar chart showing the proportions of each serotype/clonal complex in 2008–2015 and 2016–2023. Other includes the following: Ia/CC1, Ia/singleton, II/CC1, II/CC12, III/CC17, III/CC283, III/CC452, IV/CC1, IV/CC459, VIII/CC1, and NT/CC1

The relationships between serotype and CC profiles are summarized in Table 2; Figs. 1 and 2. Among the 74 GBS strains recovered from blood cultures of non-pregnant adult patients, the most common lineage was serotype Ib/CC12 (*n* = 15, 20.3%), followed by serotypes V/CC1 (*n* = 12, 16.2%) and Ia/CC23 (*n* = 7, 9.5%).

**Table 2:** Distribution of serotypes and clonal complexes among strains in this study

CC	No. of strain	Serotype										
		Ia	Ib	II	III	IV	V	VI	VII	VIII	IX	NT
1	29	1	4	2	0	1	12	5	0	2	0	2
12	17	0	15	2	0	0	0	0	0	0	0	0
17	1	0	0	0	1	0	0	0	0	0	0	0
19	9	0	0	0	5	0	4	0	0	0	0	0
23	7	7	0	0	0	0	0	0	0	0	0	0
26	3	0	0	0	0	0	3	0	0	0	0	0
283	1	0	0	0	1	0	0	0	0	0	0	0
452	5	0	0	0	1	4	0	0	0	0	0	0
459	1	0	0	0	0	1	0	0	0	0	0	0
Singleton	1	1	0	0	0	0	0	0	0	0	0	0

*CC,* clonal complex, CCs were assigned according to the PubMLST database (https://pubmlst.org/organisms/streptococcus-agalactiae) *; NT,* non-typeable

Stratification by study period revealed marked temporal shifts in lineage distribution. Between 2008 and 2015, serotype Ib/CC12 was predominant (37.5%, 9/24), whereas serotypes III/CC19, V/CC1, V/CC26, and VI/CC1 were detected in 8.3% (2/24) of the strains. In contrast, from 2016 to 2023, V/CC1 emerged as the predominant lineage (20.0%, 10/50), followed by serotypes Ia/CC23 (14.0%, 7/50), Ib/CC12 (12.0%, 6/50), and IV/CC452 (8.0%, 4/50).

Comparative analysis revealed a significant decline in serotype Ib/CC12 between 2008 and 2015 and between 2016 and 2023 (37.5% vs. 12.0%, *p* = 0.015). In contrast, the proportions of Ia/CC23 (0% vs. 14.0%, *p* = 0.088) and V/CC1 (8.3% vs. 20.0%, *p* = 0.32) increased, although the changes were not statistically significant. Notably, all seven serotype Ia/CC23 strains and 10 (83.3%) V/CC1 strains were recovered after 2016, indicating an expansion of these lineages in the last ten years. All serotype IV/CC452 strains were identified exclusively in 2016.

Among the 11 monomicrobial STSS strains, serotype Ia/CC23, Ib/CC12, III/CC19, and V/CC12 were the most frequent, each accounting for two (18.2%) strains.

### Antimicrobial susceptibility, resistance genes, and potentially endemic subclusters

All the strains were susceptible to PEN and CTX. One strain, GBSIR0652, exhibited a PEN MIC of 0.125 µg/mL and belonged to serotype III/ST464 (CC452). This strain harbored the amino acid substitutions I377V, G398A, Q412L, and H438Y in PBP2X. It exhibited resistance to ERY, CLI, and LVX and harbored the resistance determinants *ermB* and S81L in GyrA and S79F in ParC.

Resistance gene profiles of the GBS strains are presented in Table 3; Fig. 1B. Resistance rates to LVX, ERY, and CLI were 32.4%, 23.0%, and 17.6%, respectively. All strains carrying *ermB* (*n* = 12; 16.2%) were resistant to both ERY and CLI, with serotypes V/CC1 (*n* = 4; 30.0%) and III-CC19 (*n* = 3; 25.0%). Five (6.8%) strains harbored *ermA*, mainly serotypes Ib-CC12 (*n* = 2, 40.0%) and III-CC19 (*n* = 2, 40.0%). Two (2.7%) strains, Ia-CC23 and V-CC19, harbored *mefA*/*msrD*. Among the 24 LVX-resistant strains, 22 (91.6%) exhibited double substitutions in GyrA (S81L) and ParC (S79F/Y), with serotypes Ib-CC12 (*n* = 14, 58.3%) and Ib-CC1 (*n* = 4, 16.6%) being dominant.

**Table 3:** Distribution of penicillin-binding protein 2X types, resistance genes, and amino acid substitutions by serotype, clonal complex, and sequence type

Serotype (*n*)	Clonal complex (*n*)	ST (*n*)	PBP2X profile (*n*)	Macrolide resistance genes (*n*)	Tetracycline resistance genes (*n*)	Quinolone resistance substitutions (*n*)
Ia (9)	1 (1)	989 (1)	1 (1)	none (1)	*tetM* (1)	none (1)
	23 (7)	144 (4)	5 (4)	none (4)	*tetM* (4)	none (4)
		23 (3)	5 (3)	*mefA* (1), none (2)	*tetM* (3)	none (3)
	Singleton (1)	4 (1)	4 (1)	*ermB* (1)	*tetM* (1)	none (1)
Ib (19)	1 (4)	3 (4)	1 (4)	none (4)	none (4)	*gyrA*_S81L + *parC*_S79F (4)
	12 (15)	10 (14)	1 (14)	*ermB* (2), *ermA* (1), none (11)	*tetM* (2), *tetO* (1), none (11)	*gyrA*_S81L + *parC*_S79F (13), none (1)
		2328 (1)	1 (1)	none (1)	none (1)	*gyrA*_S81L + *parC*_S79F (1)
II (4)	1 (2)	1 (2)	1 (2)	none (2)	*tetM* (2)	none (2)
	12 (2)	12 (2)	1 (2)	none (2)	*tetO* (1), none (1)	none (2)
III (8)	17 (1)	17 (1)	2 (1)	none (1)	*tetM* (1)	none (1)
	19 (5)	27 (3)	1 (3)	*ermB* (3)	*tetO* (3)	none (3)
		335 (2)	1 (2)	*ermA* (2)	*tetM* (2)	none (2)
	283 (1)	283 (1)	1 (1)	none (1)	*tetM* (1)	*gyrA*_S81L + *parC*_S79Y (1)
	452 (1)	464 (1)	new_seq (1)	*ermB* (1)	none (1)	*gyrA*_S81L + *parC*_S79F (1)
IV (6)	1 (1)	3 (1)	1 (1)	none (1)	none (1)	*gyrA*_S81L + *parC*_S79F (1)
	452 (4)	452 (4)	5 (4)	none (1)	none (1)	none (1)
	459 (1)	196 (1)	1 (1)	none (1)	*tetM* (1)	none (1)
V (19)	1 (12)	1 (12)	1 (12)	*ermB* (4)	*tetM* (11)	*parC*_S79F (1)
	19 (4)	19 (4)	1 (4)	*ermA* + *mefA* (1), *ermB* (1)	*tetM* (3), *tetM* + *tetO* (1)	*gyrA*_S81L + *parC*_S79F (2)
	26 (3)	26 (3)	4 (3)	none (3)	none (3)	none (3)
VI (5)	1 (5)	1 (5)	1 (4), new_seq (1)	none (5)	none (5)	*gyrA*_S81L (1)
VIII (2)	1 (2)	1 (1)	1 (1)	none (1)	none (1)	none (1)
		2401 (1)	1 (1)	none (1)	none (1)	none (1)
NT** (2)	1 (2)	1 (2)	1 (2)	none (2)	*tetM* (1), none (1)	none (2)

*Resistance genes were detected using ABRicate with reference to the National Center for Biotechnology Information and ResFinder databases (https://www.ncbi.nlm.nih.gov/bioproject/PRJNA313047). The thresholds for coverage and identity were set at ≥ 95% **NT, non-typeable

### Vaccine target coverage estimations

The distribution of vaccine candidates is summarized in Table 4. Among all the strains, 48.6% and 87.8% were covered by trivalent (serotypes Ia, Ib, and III) and hexavalent (Ia, Ib, and II–V) polysaccharide vaccines, respectively. The coverage rate of the GBS-NN/NN2 vaccine, which targets the N-terminal domains of Alp1, Alp2/3, AlphaC, and Rib, was 94.5%. Notably, all III-ST27 strains (*n* = 3) tested negative for Srr1 and Srr2.

**Table 4:** Distribution of vaccine candidates by serotype, clonal complex, and sequence type

Serotype (*n*)	Clonal complex (*n*)	ST (*n*)	No. of positive strains					Srr (*n*)	Pilus profile (*n*)
			HvgA	Alp1	Alp2/3	AlphaC	Rib		
Ia (9)	1 (1)	989 (1)	0	0	1	0	0	1 (1)	1–2 A (1)
	23 (7)	144 (4)	0	0	0	0	4	1 (4)	2 A (4)
		23 (3)	0	2	0	0	0	1 (3)	2 A (3)
	Singleton (1)	4 (1)	0	1	0	0	0	1 (1)	1-2B (1)
Ib (19)	1 (4)	3 (4)	0	0	0	4	0	1 (4)	1–2 A (4)
	12 (15)	10 (14)	0	0	0	14	0	1 (14)	1–2 A (14)
		2328 (1)	0	0	0	1	0	1 (1)	1–2 A (1)
II (4)	1 (2)	1 (2)	0	0	2	0	0	1 (2)	1–2 A (2)
	12 (2)	12 (2)	0	0	0	2	0	1 (2)	2 A (1), 1–2 A (1)
III (8)	17 (1)	17 (1)	1	0	0	0	1	2 (1)	1-2B (1)
	19 (5)	27 (3)	0	0	0	0	3	None (3)	1–2 A (3)
		335 (2)	0	0	0	0	2	1 (2)	1–2 A (2)
	283 (1)	283 (1)	0	0	0	1	0	1 (1)	1–2 A (1)
	452 (1)	464 (1)	0	0	1	0	0	1 (1)	1–2 A (1)
IV (6)	1 (1)	3 (1)	0	0	0	1	0	1 (1)	1–2 A (1)
	452 (4)	452 (4)	0	0	0	4	0	2 (4)	2B (4)
	459 (1)	196 (1)	0	1	0	0	0	1 (1)	1–2 A (1)
V (19)	1 (12)	1 (12)	0	0	12	0	0	1 (12)	1–2 A (12)
	19 (4)	19 (4)	0	3	0	0	0	1 (4)	1–2 A (4)
	26 (3)	26 (3)	0	0	0	1	0	1 (3)	2 A (3)
VI (5)	1 (5)	1 (5)	0	0	0	5	0	1 (5)	1–2 A (5)
VIII (2)	1 (2)	1 (1)	0	0	1	0	0	1 (1)	1-2B (1)
		2401 (1)	0	0	1	0	0	1 (1)	1-2B (1)
NT* (2)	1 (2)	1 (2)	0	0	1	1	0	1 (2)	1–2 A (2)

*NT, non-typeable

### Pairwise SNP distance of strains in the present study and previous pediatric iGBS strains in Japan

Among the two ST464 strains, the pairwise SNP distance was ≤ 15 (specifically, 13), indicating a genetic linkage between the two strains (Tables S1–S13, Figs. S1–S11). One strain (GBSIR0652) was obtained from an adult male in January 2009 and was sequenced in the present study. The other strain (GBSIRa0060) was recovered from the blood of a 50-day-old infant from the same region in September 2010 and was sequenced in a previous study [12]. Additionally, one ST23 and two ST452 strains from the present study exhibited potential genetic linkages with strains collected in a previous pediatric iGBS surveillance study, with pairwise SNP distances ranging from 16 to 20 (Tables S1–S13). A separate set of strains from the same patient (GBSIR0707 and GBSIR0709) showed an SNP distance of zero. A 60-year-old man initially developed GBS bacteremia secondary to cellulitis and achieved clinical resolution following appropriate antimicrobial therapy. Four months later, the patient developed cellulitis at the same anatomical site, which was complicated by GBS bacteremia. Both strains belonged to Ib/CC1.

Detailed information on the strains analyzed in this study is provided in the Supplementary Data.

## Discussion

In this study, among the 74 GBS strains isolated from blood cultures of non-pregnant adult patients, the most common lineage was Ib/CC12, followed by V/CC1 and Ia/CC23. Notably, although not statistically significant, our findings indicated an expansion of the Ia/CC23 and V/CC1 lineages since 2016 among invasive GBS strains from non-pregnant adults. The predominant molecular serotypes and CC distribution observed during 2008–2015 were consistent with the results of previous studies on GBS-causing invasive adult infections in Japan [9, 10]. By contrast, the serotype and CC distributions observed in our study differ from those reported in previous Japanese studies of GBS strains obtained from vaginal samples of pregnant women and infants with invasive infections, in which III/CC17, Ia/CC23, and III/CC19 were the major circulating lineages [12, 24, 25].

In this study, other bacterial species were detected in the blood cultures of approximately 18% of patients with GBS bacteremia. The most frequently co-isolated species was *Staphylococcus aureus*, followed by *Escherichia coli*. Several previous studies on iGBS in non-pregnant adults have suggested that polymicrobial bacteremia may be associated with poorer outcomes [26, 27]. However, whether the co-detected bacterial species directly interact with GBS to enhance its virulence remains unclear, and the STSS observed in previous studies on iGBS may reflect pathophysiological effects driven by organisms other than GBS. Comprehensive metaomics approaches such as metatranscriptomics may provide deeper insights into these interactions.

In this study, six serotype IV GBS strains were identified: ST452/CC452 (*n* = 4), ST3/CC1 (*n* = 1), and ST196/CC459 (*n* = 1). Serotype IV/ST452 GBS was initially detected at our institution in 2018. An increasing prevalence of serotype IV/CC452 has been documented in the United States and Canada since the early 2000s [28, 29]. However, previous Japanese studies focusing on iGBS infections in adults did not identify this lineage [9, 10]. By contrast, more recent studies from Japan have demonstrated the emergence of IV/CC452 among pregnant women and children [12, 16]. Kasai et al.. analyzed invasive pediatric GBS infections between 2004 and 2023, and reported that IV/CC452 was first detected in 2019 [12]. Collectively, these findings suggest that serotype IV/CC452 GBS has been increasingly circulating in Japan in recent years, affecting pregnant women, children, and adults.

We identified one serotype III/ST464 (CC452) strain exhibiting a high MIC for PCG. In Japan, serotype III/ST464 (CC452) PEN-resistant GBS (PRGBS), with the same amino acid substitutions as our strain, has been previously reported [1, 30]. PRGBS strains in Japan have most commonly been recovered from respiratory specimens of older patients, with serotype VI/ST1 or ST458 (a single-locus variant of ST1 within CC1) commonly associated with PRGBS [1, 30–33]. Although only a single strain with reduced susceptibility to PEN was detected in this study, the World Health Organization has classified PRGBS as a medium-priority pathogen in its 2024 bacterial priority pathogens list [34], underscoring the need for ongoing surveillance and research.

In our study, the resistance rates to ERY and CLI were 22.6% and 17.3%, respectively, which were lower than those reported for GBS strains from Japanese children with iGBS (61.2% and 43.5%, respectively) [12]. This difference is likely explained by the fact that nearly half of the pediatric strains were serotype III, the majority of which belonged to ST17. In Japan, serotype III/ST17 strains typically harbor *ermB* and show resistance to both ERY and CLI; however, only one such strain was identified in our collection.

In this study, alpha-like protein-based GBS-NN/NN2 and hexavalent polysaccharide vaccines demonstrated high coverage rates, exceeding 90%, whereas the trivalent polysaccharide vaccine showed substantially lower coverage (48.6%). For comparison, the reported coverage rates of trivalent and hexavalent polysaccharide GBS vaccines against iGBS in children in Japan from 2004 to 2023 were 87.8% and 98.3%, respectively [12]. The lower coverage of the trivalent vaccine observed in this study is likely attributable to the increasing prevalence of V/CC1 strains in recent years.

A previous study on pediatric iGBS provided evidence of persistent regional circulation, particularly within the ST17 and ST23 lineages [12]. Among the strains collected in the present study, one strain differed by 13 SNPs from a strain previously reported in a pediatric patient treated at a different hospital in the same prefecture (Hiroshima Prefecture), suggesting a possible epidemiological link within a limited geographic setting. In addition, although the SNP distances were somewhat greater in the three other cases (16–20 SNPs), these findings also suggested the possibility of transmission across regional and population boundaries. Taken together, these observations are consistent with previous reports and suggest that clonally related GBS strains may be shared across different populations within a limited geographic setting, although this should be interpreted with caution given the scope of this single-center study. Further studies using larger and more representative datasets are required to clarify potential transmission pathways and their relevance to prevention strategies.

In the patient with two episodes of GBS bacteremia, SNP analysis showed that strains from both episodes were genomically identical. However, the mechanism underlying these episodes remains unclear due to the lack of data on colonization and strain genotyping.

This study had several limitations. It was conducted at a single tertiary care center and included a relatively small number of patients. Therefore, these findings should not be interpreted as representative of the nationwide epidemiology of iGBS in Japan. Moreover, the distributions of GBS serotypes, genotypes, clinical features, and patient demographics observed in this cohort may not fully reflect those reported in other countries. Larger, multicenter, or nationwide surveillance studies are warranted to validate and generalize these observations.

In conclusion, we clarified the rate of STSS and 30-day mortality among non-pregnant adults with iGBS at a single center in Japan and performed molecular characterization of 74 GBS strains. These results suggest a recent expansion of the Ia/CC23 and V/CC1 lineages in this population and indicate that the trivalent polysaccharide vaccine provides relatively low coverage. Our SNP analysis identified several adult iGBS strains that differed by no more than 20 SNPs from strains previously reported in pediatric iGBS cases in Japan. This finding suggests that GBS may be transmitted not only through the well-recognized route of vertical mother-to-child transmission but also via other transmission pathways, thereby potentially contributing to invasive infections in distinct populations. Although direct transmission routes cannot yet be conclusively determined, genomic analysis of GBS strains collected from multiple clinical and ecological sources may contribute to a better understanding of population structure and possible epidemiological links relevant to future prevention strategies.

## Supplementary Information

Below is the link to the electronic supplementary material.

Supplementary Material 1

Supplementary Material 2

Supplementary Material 3

## Data Availability

The whole-genome sequencing data generated in this study are available under BioProject accession number PRJDB35542.
