# Supplementary material for: Clinical and molecular characteristics of Streptococcus agalactiae bacteremia in nonpregnant adults: a single-center analysis over 16 years in Hiroshima, Japan
Source: Eur J Clin Microbiol Infect Dis. 2026 Apr 30;45(8):2471–83. doi: 10.1007/s10096-026-05520-6 (PMC13428702; doi:10.1007/s10096-026-05520-6)
Supplement: Supplementary file 3 — Supplementary Material 3 [file 10096_2026_5520_MOESM3_ESM.docx]

Clinical and molecular characteristics of *Streptococcus agalactiae* bacteremia in nonpregnant adults: a single-center analysis over a 16-year period in Hiroshima, Japan

Supplementary figures


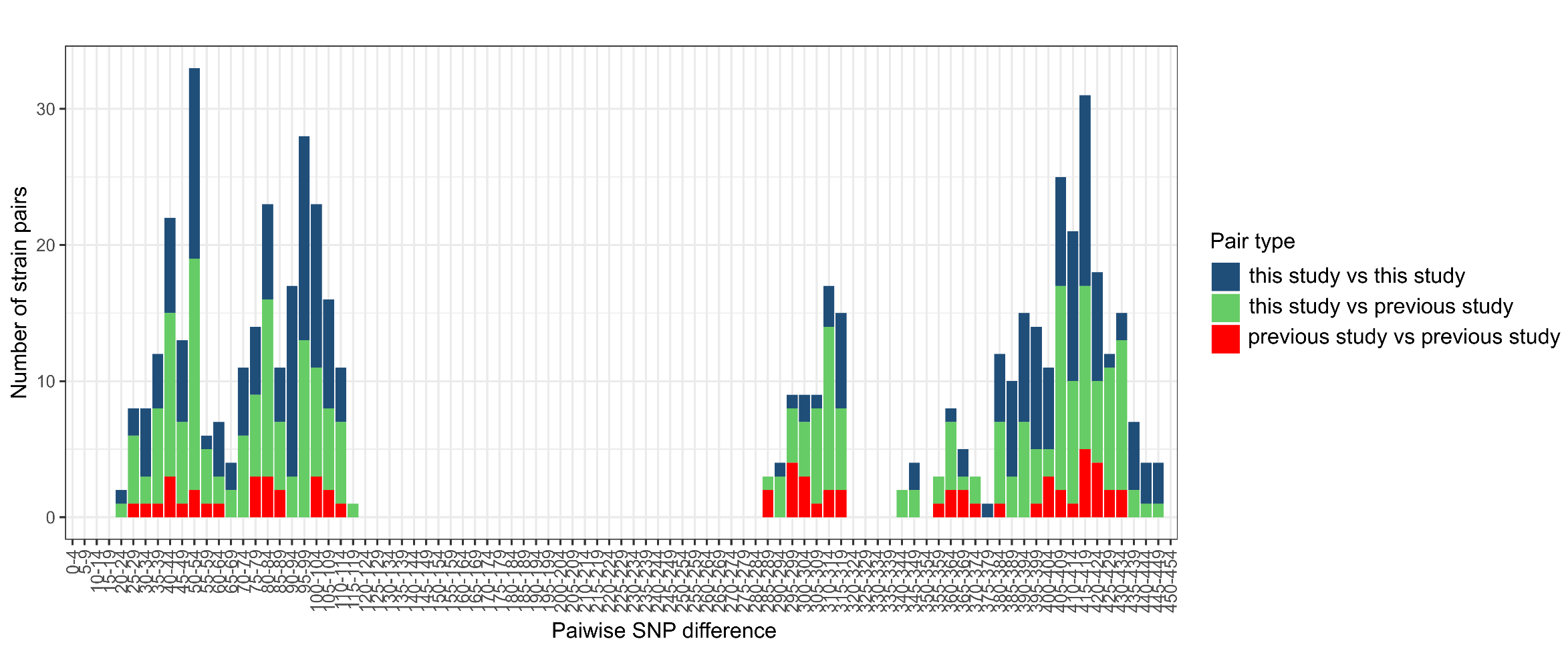


Figure S1. Integrated pairwise SNP analysis of ST1 strains detected in this study and strains reported by Kasai *et al*. (J Infect Dis. 2026 Jan 17;233(1):e11-e21.) from pediatric patients with invasive GBS infection in Japan between 2004 and 2023. Histograms show the number of strain sets included within each pairwise SNP range. Blue indicates pairs of strains detected in this study, green indicates pairs consisting of one strain from this study and one reported by Kasai *et al.*, and red indicates pairs of strains reported by Kasai *et al.*


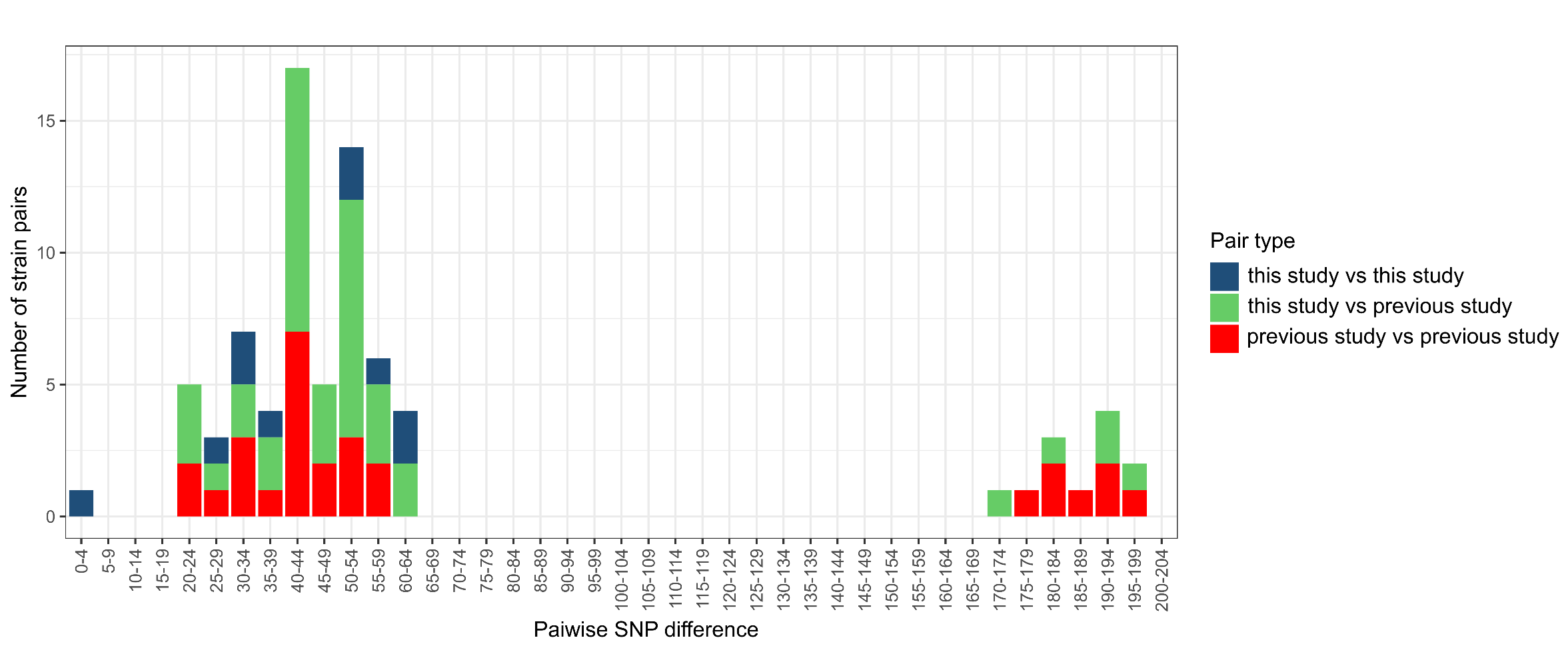


Figure S2. Integrated pairwise SNP analysis of ST3 strains detected in this study and strains reported by Kasai *et al*. (J Infect Dis. 2026 Jan 17;233(1):e11-e21.) from pediatric patients with invasive GBS infection in Japan between 2004 and 2023. Histograms show the number of strain sets included within each pairwise SNP range. Blue indicates pairs of strains detected in this study, green indicates pairs consisting of one strain from this study and one reported by Kasai *et al.*, and red indicates pairs of strains reported by Kasai *et al.*


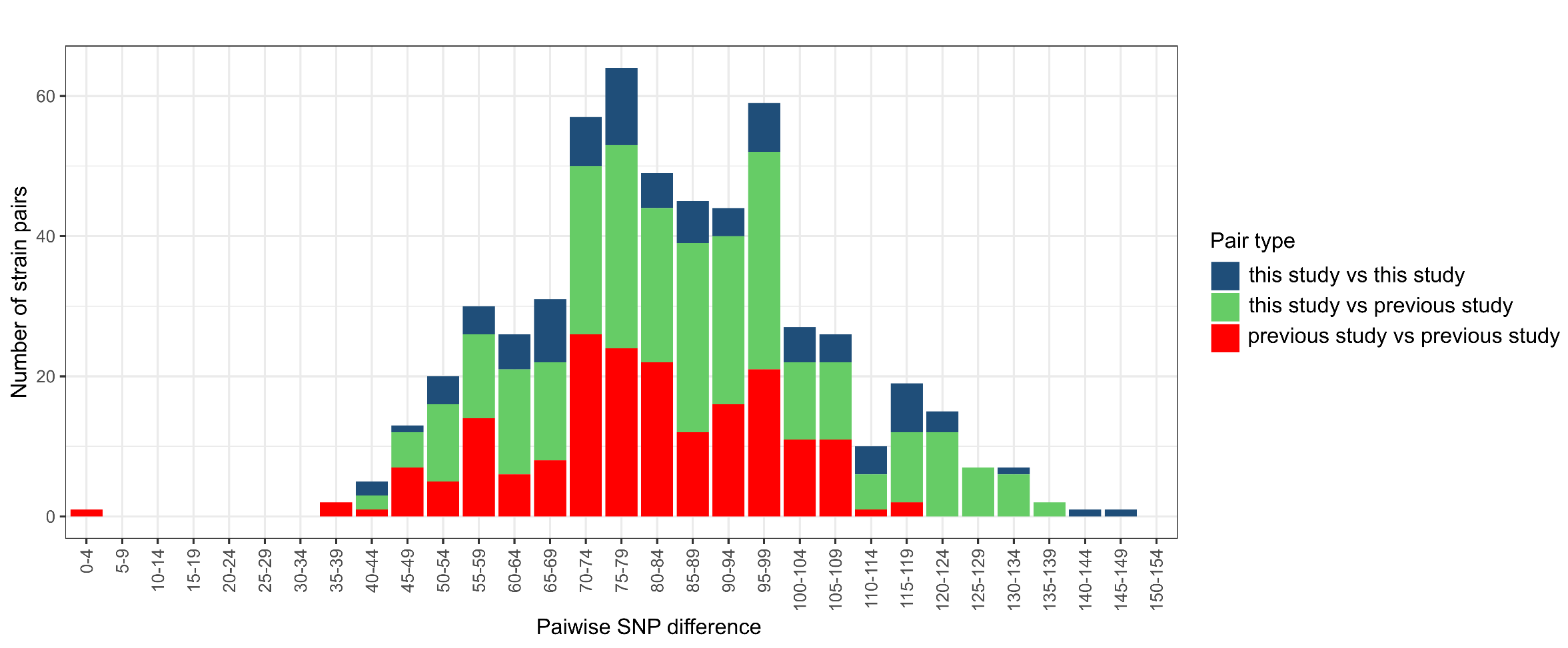


Figure S3. Integrated pairwise SNP analysis of ST10 strains detected in this study and strains reported by Kasai *et al.* (J Infect Dis. 2026 Jan 17;233(1):e11-e21.) from pediatric patients with invasive GBS infection in Japan between 2004 and 2023. Histograms show the number of strain sets included within each pairwise SNP range. Blue indicates pairs of strains detected in this study, green indicates pairs consisting of one strain from this study and one reported by Kasai *et al.*, and red indicates pairs of strains reported by Kasai *et al.*


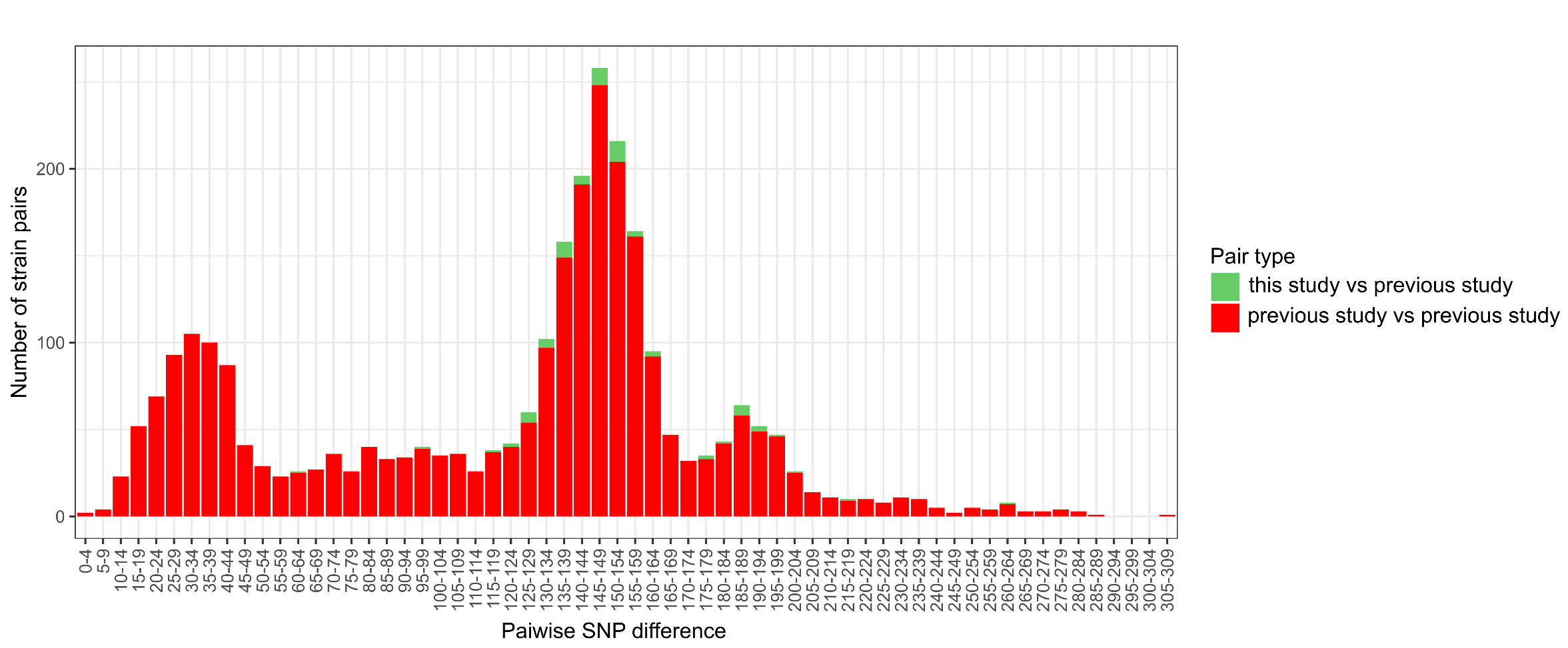


Figure S4. Integrated pairwise SNP analysis of ST17 strains detected in this study and strains reported by Kasai *et al.* (J Infect Dis. 2026 Jan 17;233(1):e11-e21.) from pediatric patients with invasive GBS infection in Japan between 2004 and 2023. Histograms show the number of strain sets included within each pairwise SNP range. Blue indicates pairs of strains detected in this study, green indicates pairs consisting of one strain from this study and one reported by Kasai *et al.*, and red indicates pairs of strains reported by Kasai *et al.*


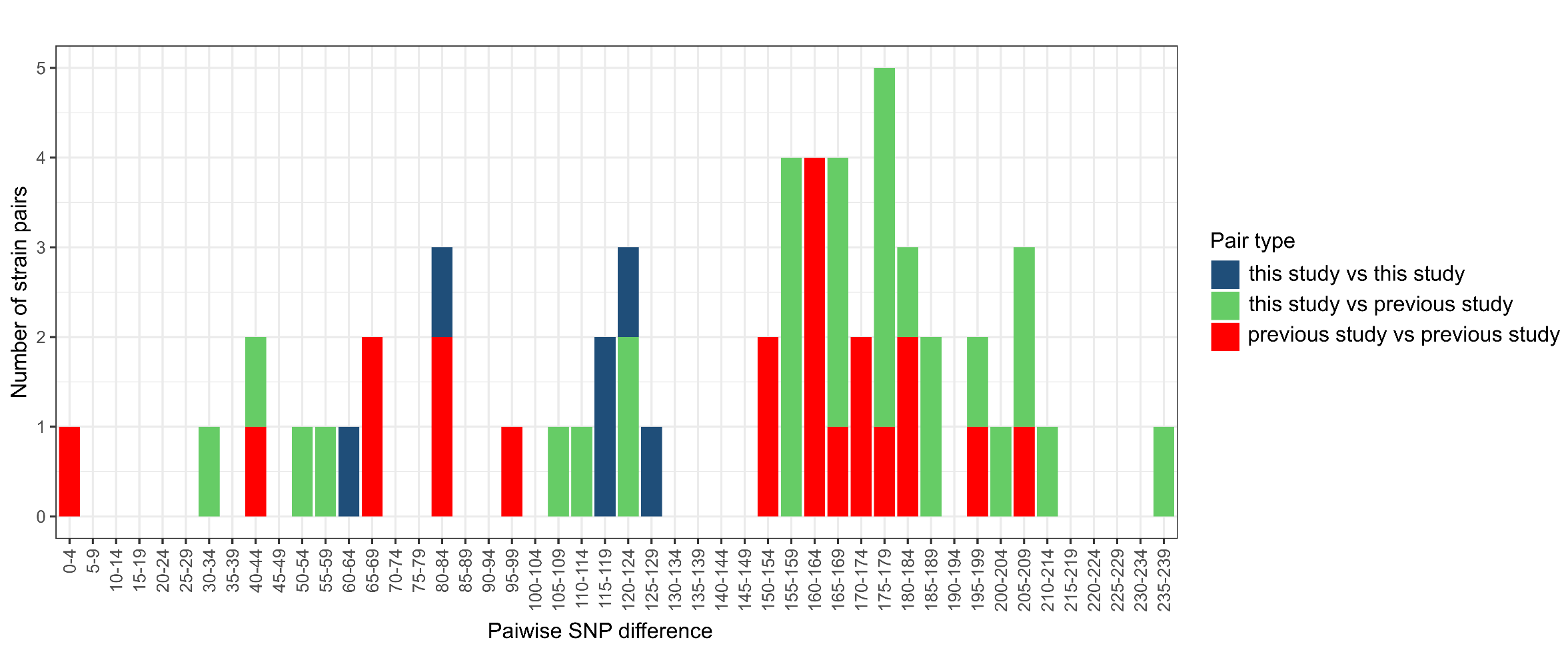


Figure S5. Integrated pairwise SNP analysis of ST19 strains detected in this study and strains reported by Kasai *et al.* (J Infect Dis. 2026 Jan 17;233(1):e11-e21.) from pediatric patients with invasive GBS infection in Japan between 2004 and 2023. Histograms show the number of strain sets included within each pairwise SNP range. Blue indicates pairs of strains detected in this study, green indicates pairs consisting of one strain from this study and one reported by Kasai *et al.*, and red indicates pairs of strains reported by Kasai *et al.*


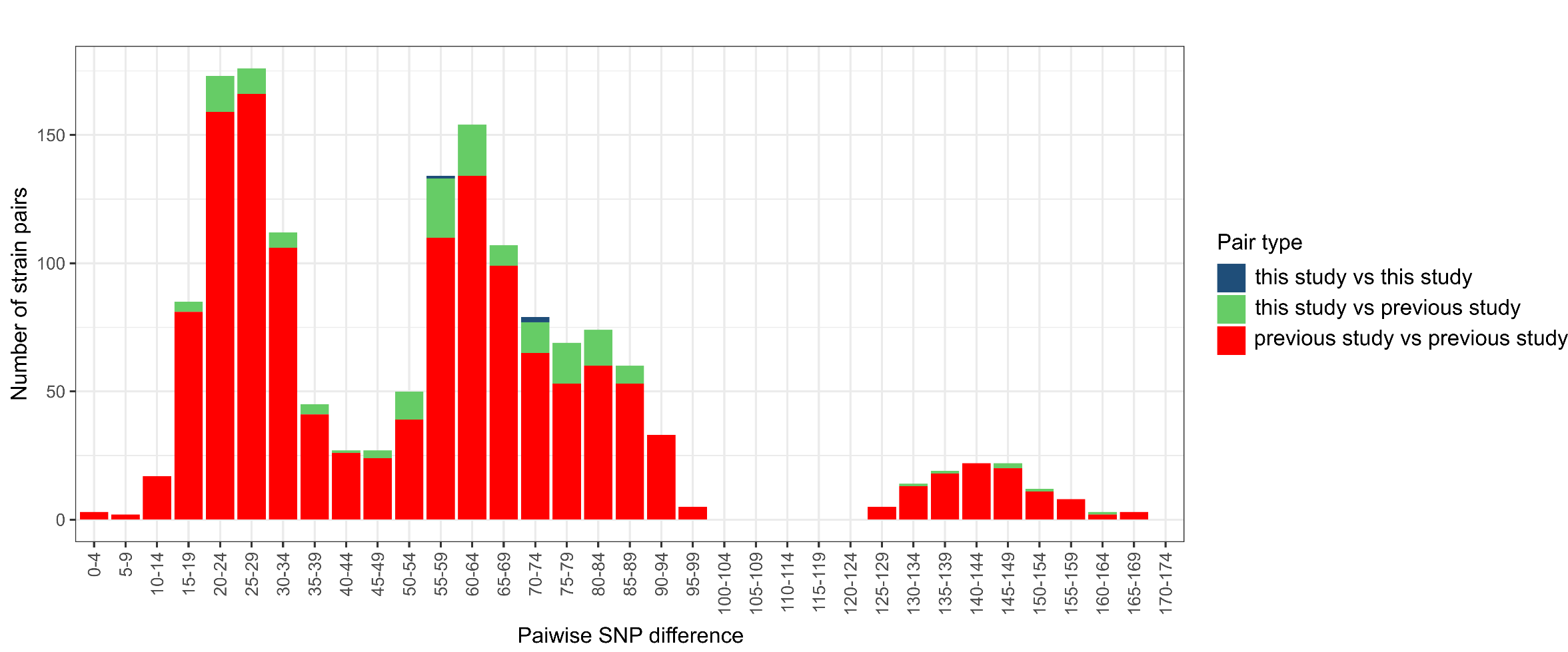


Figure S6. Integrated pairwise SNP analysis of ST23 strains detected in this study and strains reported by Kasai *et al.* (J Infect Dis. 2026 Jan 17;233(1):e11-e21.) from pediatric patients with invasive GBS infection in Japan between 2004 and 2023. Histograms show the number of strain sets included within each pairwise SNP range. Blue indicates pairs of strains detected in this study, green indicates pairs consisting of one strain from this study and one reported by Kasai *et al.*, and red indicates pairs of strains reported by Kasai *et al.*


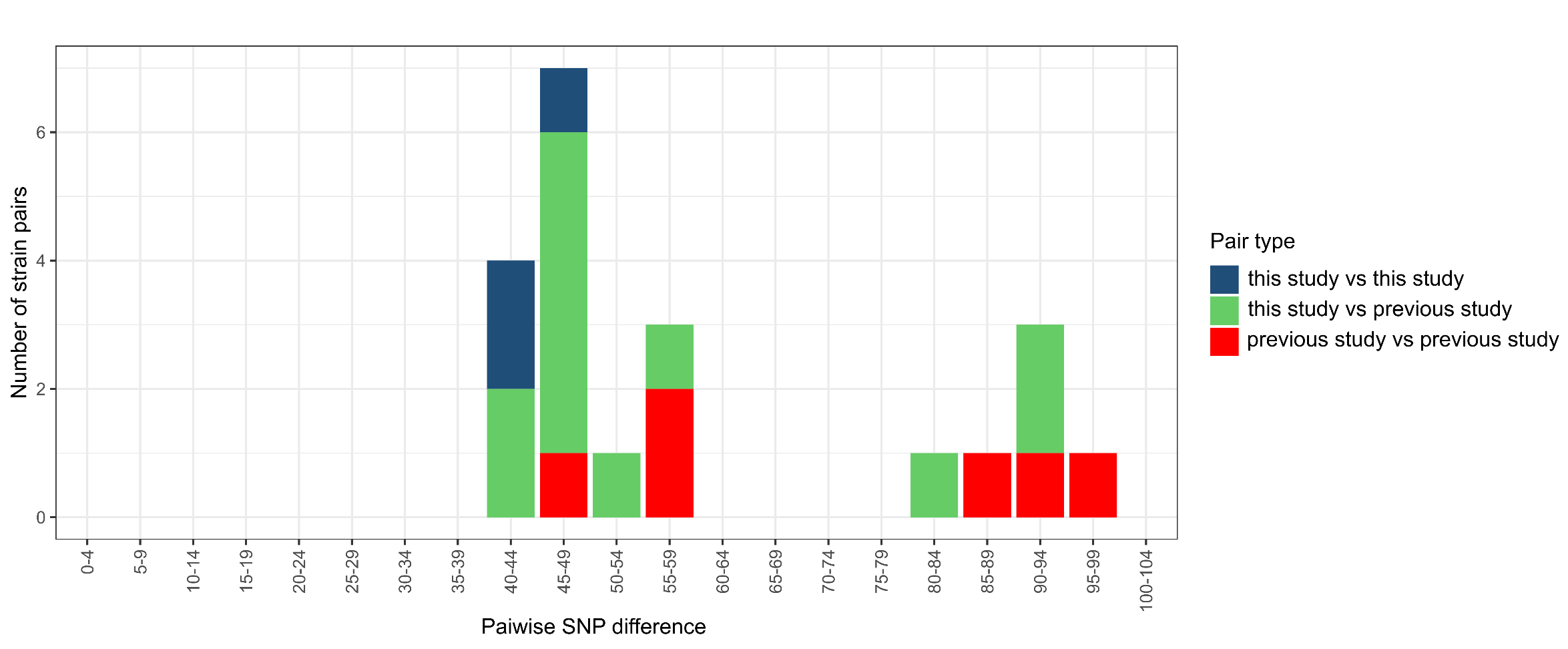


Figure S7. Integrated pairwise SNP analysis of ST26 strains detected in this study and strains reported by Kasai *et al.* (J Infect Dis. 2026 Jan 17;233(1):e11-e21.) from pediatric patients with invasive GBS infection in Japan between 2004 and 2023. Histograms show the number of strain sets included within each pairwise SNP range. Blue indicates pairs of strains detected in this study, green indicates pairs consisting of one strain from this study and one reported by Kasai *et al.*, and red indicates pairs of strains reported by Kasai *et al.*


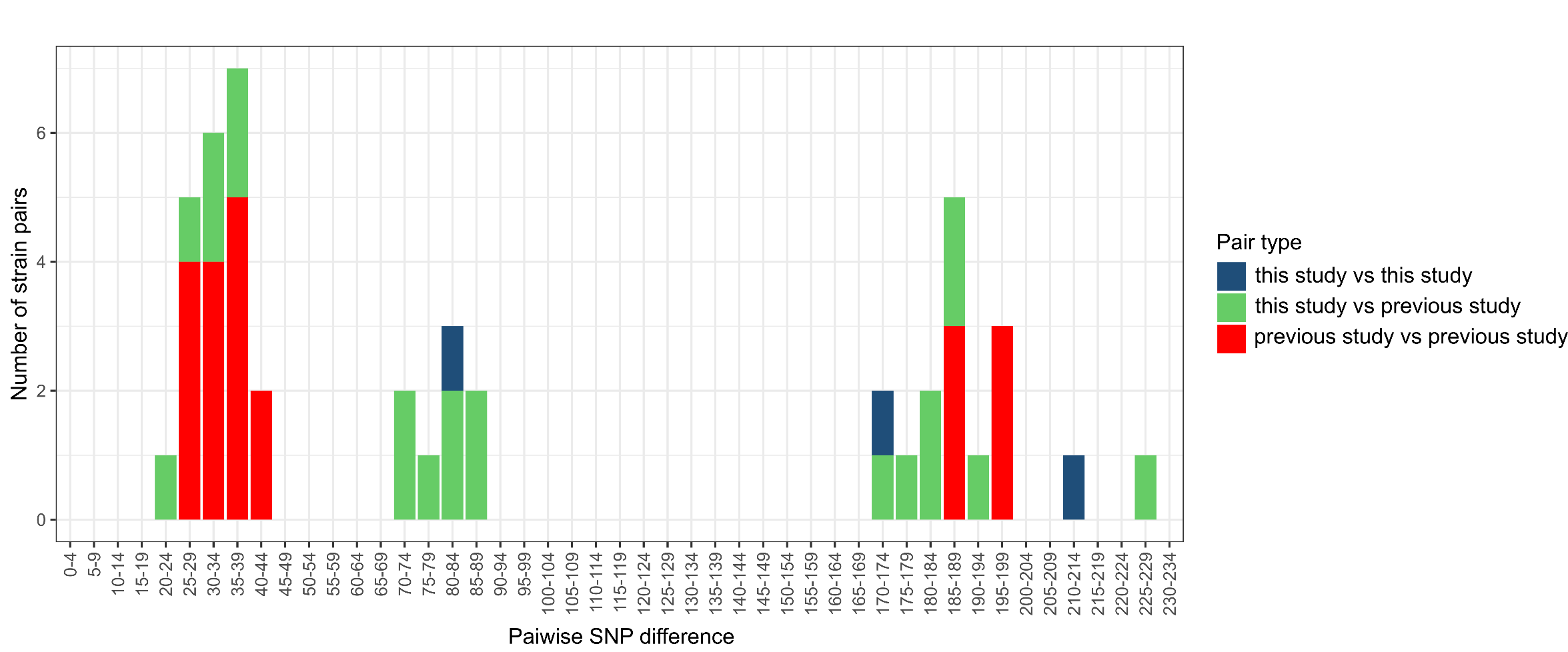


Figure S8. Integrated pairwise SNP analysis of ST27 strains detected in this study and strains reported by Kasai *et al.* (J Infect Dis. 2026 Jan 17;233(1):e11-e21.) from pediatric patients with invasive GBS infection in Japan between 2004 and 2023. Histograms show the number of strain sets included within each pairwise SNP range. Blue indicates pairs of strains detected in this study, green indicates pairs consisting of one strain from this study and one reported by Kasai *et al.*, and red indicates pairs of strains reported by Kasai *et al.*


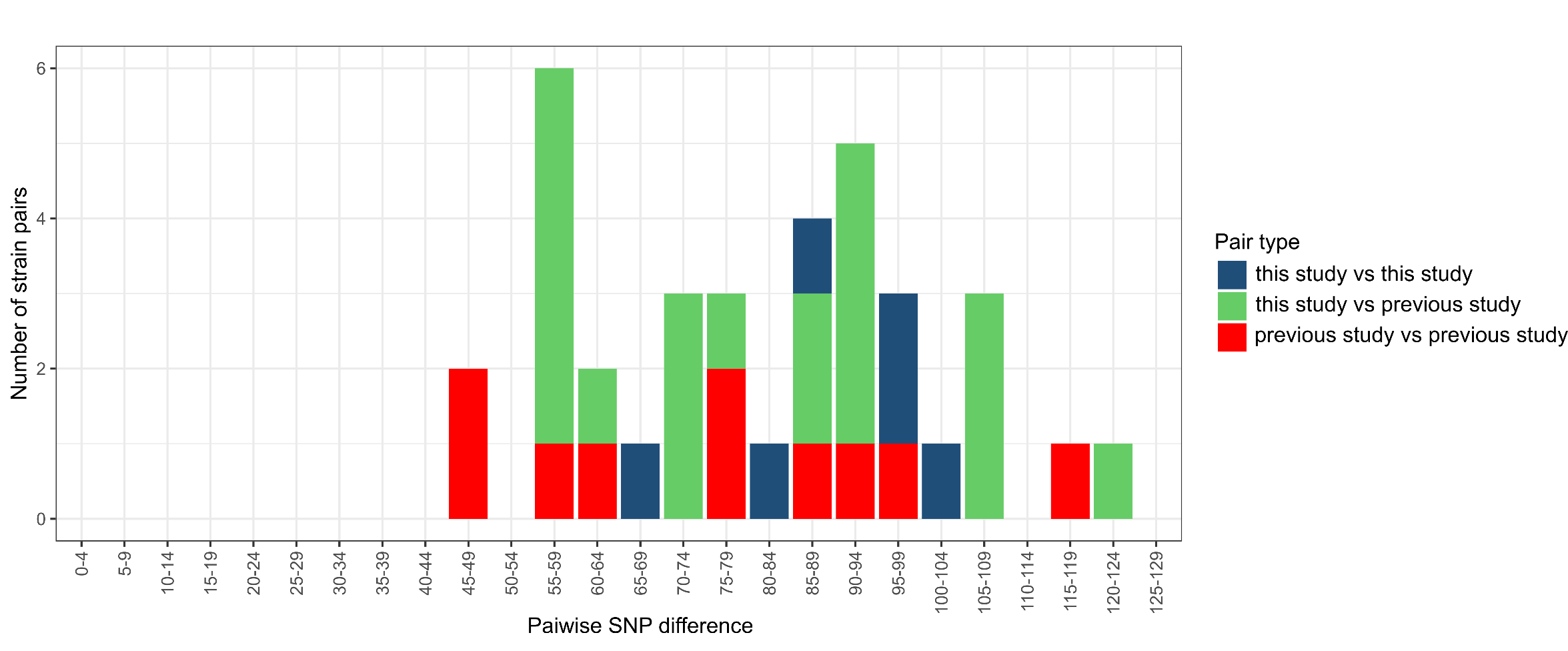


Figure S9. Integrated pairwise SNP analysis of ST144 strains detected in this study and strains reported by Kasai *et al.* (J Infect Dis. 2026 Jan 17;233(1):e11-e21.) from pediatric patients with invasive GBS infection in Japan between 2004 and 2023. Histograms show the number of strain sets included within each pairwise SNP range. Blue indicates pairs of strains detected in this study, green indicates pairs consisting of one strain from this study and one reported by Kasai *et al.*, and red indicates pairs of strains reported by Kasai *et al.*


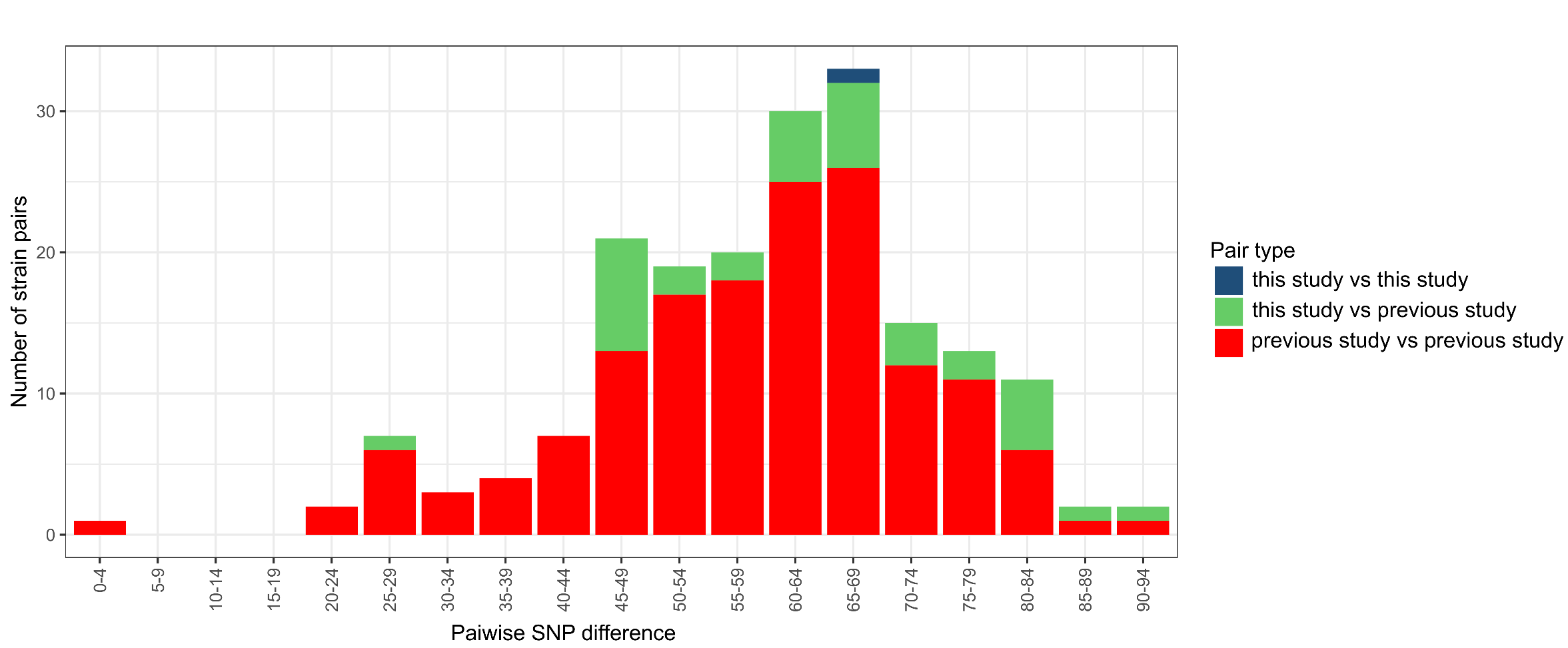


Figure S10. Integrated pairwise SNP analysis of ST335 strains detected in this study and strains reported by Kasai *et al.* (J Infect Dis. 2026 Jan 17;233(1):e11-e21.) from pediatric patients with invasive GBS infection in Japan between 2004 and 2023. Histograms show the number of strain sets included within each pairwise SNP range. Blue indicates pairs of strains detected in this study, green indicates pairs consisting of one strain from this study and one reported by Kasai *et al.*, and red indicates pairs of strains reported by Kasai *et al.*


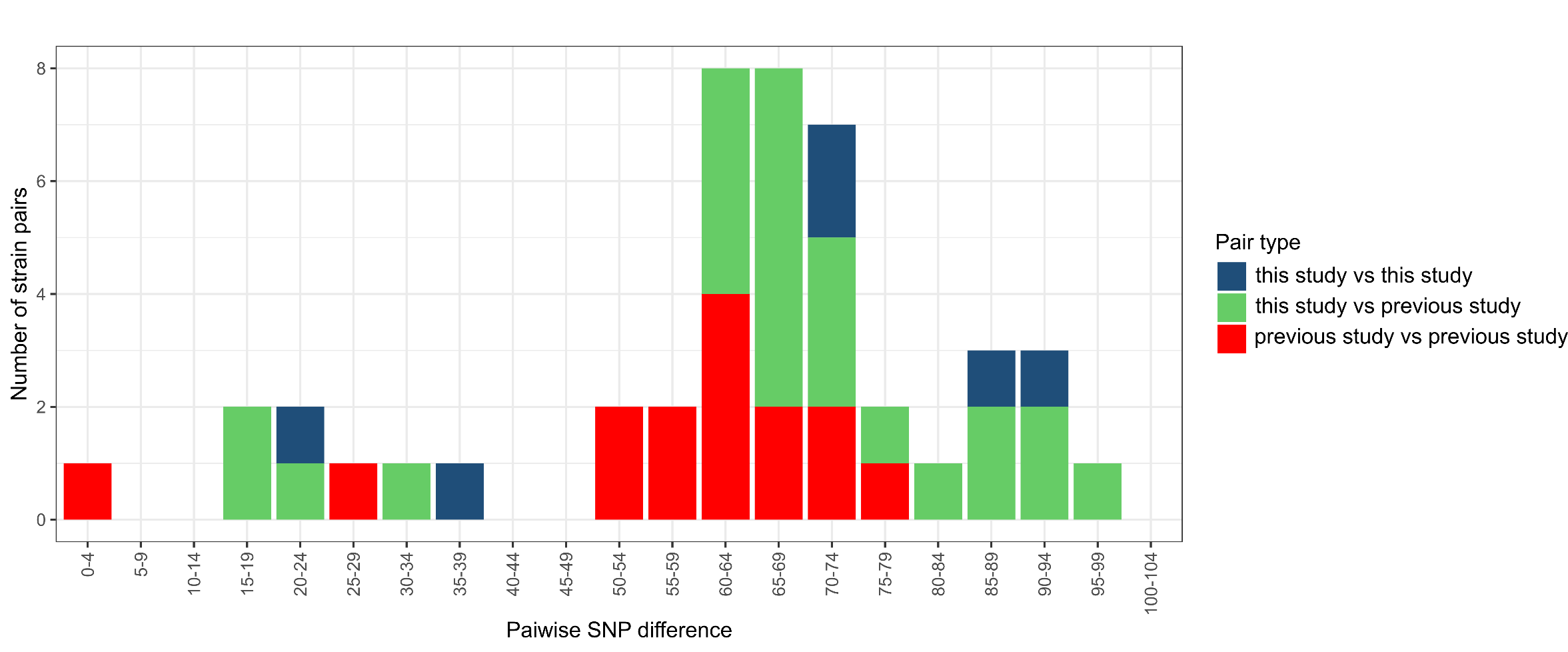


Figure S11. Integrated pairwise SNP analysis of ST452 strains detected in this study and strains reported by Kasai *et al.* (J Infect Dis. 2026 Jan 17;233(1):e11-e21.) from pediatric patients with invasive GBS infection in Japan between 2004 and 2023. Histograms show the number of strain sets included within each pairwise SNP range. Blue indicates pairs of strains detected in this study, green indicates pairs consisting of one strain from this study and one reported by Kasai *et al.*, and red indicates pairs of strains reported by Kasai *et al.*
